# Probabilistic Inference for Dynamical Systems

**DOI:** 10.3390/e20090696

**Published:** 2018-09-12

**Authors:** Sergio Davis, Diego González, Gonzalo Gutiérrez

**Affiliations:** 1 Comisión Chilena de Energía Nuclear, Casilla 188-D Santiago, Chile; 2 Departamento de Física, Facultad de Ciencias Exactas, Universidad Andres Bello, Sazié 2212, Piso 7, 8370136 Santiago, Chile; 3 Grupo de Nanomateriales, Departamento de Física, Facultad de Ciencias, Universidad de Chile, Casilla 653 Santiago, Chile

**Keywords:** dynamical systems, bayesian inference, fluid equations

## Abstract

A general framework for inference in dynamical systems is described, based on the language of Bayesian probability theory and making use of the maximum entropy principle. Taking the concept of a path as fundamental, the continuity equation and Cauchy’s equation for fluid dynamics arise naturally, while the specific information about the system can be included using the maximum caliber (or maximum path entropy) principle.

## 1. Introduction

Dynamical system models are widely used to describe complex physical systems (e.g., the weather), as well as social and economic systems (e.g., the stock market). These systems are usually subject to high levels of uncertainty, either in their initial conditions and/or in their interactions with their environment. From the point of view of constructing predictive models, the optimal description of the time-dependent state of such a system given external constraints is a challenge with promising applications in both fundamental and applied science. This is of course an *inference problem* in which we must choose the most likely solution out of the (possibly infinite) alternatives compatible with the given information we have about the system.

Given all this, it seems that a unified framework for performing inference on dynamical systems may open new possibilities in several areas, including non-equilibrium statistical mechanics and thermodynamics, hydrodynamics (including magnetohydrodynamics), and classical mechanics under stochastic forces, among other possible fields of application. Of course, this vision of inference applied to dynamical systems is not new—the clearest exposition of the ideas that we aim to extend here was given by E. T. Jaynes [1,2], followed by several others [3,4,5].

In this work, we present some elements for a general framework of inference in dynamical systems, written in the language of Bayesian probability. The first is the master equation, which is shown as a direct consequence of the laws of probability. Next, we develop the treatment of inference over paths from which we obtain the continuity equation and Cauchy’s equation for fluid dynamics, and discuss their range of applicability. Finally, we close with some concluding remarks.

## 2. Why Bayesian Inference?

Unlike the standard (“frequentist”) interpretation of probability theory, in which probabilities are frequencies of occurrence of repeatable events, Bayesian probability can be understood as the natural extension of classical logic in the case of *uncertainty* [6,7]. Bayesian probability deals with unknown quantities rather than identical repetitions/copies of an event or system, and is able to include *prior* information when needed.

The conceptual framework of Bayesian probability provides an elegant language to describe dynamical systems under uncertainty. A straightforward advantage of the Bayesian framework is that one does not need to assume an ensemble of “many identical copies of a system”. A single system with uncertain initial conditions and/or forces is sufficient to construct a theory. The probability of finding this particular system in a given state at a given time would not be a frequency, but rather a degree of plausibility conditioned on the known information. In fact, we can lift even the common assumption of “many degrees of freedom”. The motion of a single particle could be used to construct an internally consistent theory, where the equations of motion for time-dependent probability densities are similar to the ones of hydrodynamics. We will describe in detail both features of the Bayesian formulation in the following sections.

A brief overview of Bayesian notation used in this work follows. We will take P(Q|I) as the probability of a particular *proposition* Q being true given knowledge I. On the other hand, ${\langle G\rangle }_{I}$ will denote the expected value of an arbitrary *quantity* *G* given knowledge I, and will be given by

(1) $${\langle G\rangle }_{I}=\sum_{u}P\left(u|I\right)G\left(u\right),$$

where u represents one of the possible states of the system.

## 3. Dynamical Evolution of Probabilities

Consider a discrete-time system that can transit between *n* possible states $\left\{x_{1},\ldots ,x_{n}\right\}$ at different times. If we denote by X(t) the state of the system at time *t*, we have that the joint probability of being in state *a* at time *t* and in state *b* at time t+Δt is given by

(2) P(X(t)=a,X(t+Δt)=b|I)=P(X(t+Δt)=b|X(t)=a,I)·P(X(t)=a|I).

By summing over *a* in Equation (2) and taking b=x, we have

(3) $$P\left(X\left(t+\Delta t\right)=x|I\right)=\sum_{a}P\left(X\left(t+\Delta t\right)=x|X\left(t\right)=a,I\right)\cdot P\left(X\left(t\right)=a|I\right),$$

while by summing over *b* and taking a=x, we have

(4) $$P\left(X\left(t\right)=x|I\right)=P\left(X\left(t\right)=x|I\right)\sum_{b}P\left(X\left(t+\Delta t\right)=b|X\left(t\right)=x,I\right).$$

From these two identities (Equations (3) and (4)), we can construct the discrete-time difference,

(5) $$\begin{array}{cc} \frac{P(X(t+\Delta t)=x|I)-P(X(t)=x|I)}{\Delta t} & =\sum_{x^{\prime }}\left[\frac{P(X\left(t+\Delta t\right)=x|X\left(t\right)=x^{\prime },I)}{\Delta t}\right]P\left(X\left(t\right)=x^{\prime }|I\right)\\ & -P\left(X\left(t\right)=x|I\right)\sum_{x^{\prime }}\left[\frac{P(X\left(t+\Delta t\right)=x^{\prime }|X\left(t\right)=x,I)}{\Delta t}\right]. \end{array}$$

This equation is a discrete-time form of the celebrated “master equation” [8,9,10], involving time-dependent transition probabilities. The case where $P(X\left(t+\Delta t\right)=x|X\left(t\right)=x^{\prime },I)$ is independent of *t* (i.e., a function only of the initial state $x^{\prime }$ and final state *x*) is more commonly known as the master equation in the literature. In the continuous-time limit when Δt→0, we can write this equation as:

(6) $$\frac{\partial \rho }{\partial t}=\sum_{x^{\prime }}W_{t}\left(x^{\prime }\to x\right)\rho \left(x^{\prime };t\right)-\rho \left(x;t\right)\sum_{x^{\prime }}W_{t}\left(x\to x^{\prime }\right),$$

where the instantaneous density ρ is given by ρ(a;t):=P(X(t)=a|I) and we have defined the (continuous-time) *transition rate* $W_{t}$ as

(7) $$W_{t}\left(a\to b\right):=\lim_{\Delta t\to 0}\frac{P(X(t+\Delta t)=b|X(t)=a,I)}{\Delta t}.$$

In this sense, the master equation as written in Equation (5) is a direct consequence of the laws of probability, and its validity is universal whenever we have transitions between states. Please note that there is no requirement for the system to be Markovian: regardless of the form of the joint probability

$$P(X\left(t+\Delta t\right)=x,X\left(t\right)=x^{\prime },X\left(t-\Delta t\right)=x^{\prime \prime },\ldots |I),$$

there is always a *marginal* model

(8) $$P\left(X\left(t+\Delta t\right)=x,X\left(t\right)=x^{\prime }|I\right)=\int dx^{\prime \prime }\ldots P\left(X\left(t+\Delta t\right)=x,X\left(t\right)=x^{\prime },X\left(t-\Delta t\right)=x^{\prime \prime },\ldots |I\right)$$

which yields the transition probability in Equation (7) as:

(9) $$P\left(X\left(t+\Delta t\right)=x|X\left(t\right)=x^{\prime },I\right)=\frac{P(X\left(t+\Delta t\right)=x,X\left(t\right)=x^{\prime }|I)}{\int d\eta P(X\left(t+\Delta t\right)=\eta ,X\left(t\right)=x^{\prime }|I)}.$$

It is for this probability that Equation (5) holds. In general, the transition rate $W_{t}$ will most probably be time-dependent, due to the fact that it captures the dependence of the previous history of the system up to *t*. It follows from this that all probabilities of time-dependent quantities must evolve in time according to Equation (5) (or (6) in the case of continuous time) for some (possibly time-dependent) transition probability (rate). This continuous-time master equation (Equation (6)) is more general than the continuity equation, as it includes the case where some quantities can be created or destroyed during a process. However, time evolution under global and local conservation laws is a fundamental case that can also be readily obtained from the Bayesian formalism, as we will see in the following sections. As is well-known, the continuous-time master equation can be approximated in the limit of infinitesimally small transitions to obtain the Fokker–Planck equation [9,11], but in the next section we start from the existence of continuous paths as a postulate.

## 4. Fluid Theories in a Bayesian Formulation

We will now consider a dynamical system that follows a path X(t)∈X in time, where X denotes the space of all paths consistent with given boundary conditions. The path X(t) is not completely known, and we only have access to partial information denoted by I.

In this setting, Bayesian theory defines a functional P[X|I] that is the probability density of the path X(t) being the “true path” under the known information. For any arbitrary *functional* F[X] of the path, we can then write its expected value as a path integral:

(10) $${\langle F\rangle }_{I}=\int_{X}DXP\left[X|I\right]F\left[X\right].$$

On the other hand, the expected value of any instantaneous *quantity* A(x;t) is given by

(11) $${\langle A\rangle }_{t,I}=\int_{X}DXP\left[X|I\right]A\left(X\left(t\right);t\right)=\int_{V}dx\rho \left(x;t\right)A\left(x;t\right),$$

where ρ(x;t):=P(X(t)=x|I) is the instantaneous probability density at time *t*.

By using a quantity $A\left(x^{\prime };t\right)=\delta \left(x^{\prime }-x\right)$, we see that the probability density itself has a path integral representation:

(12) $$\rho \left(x;t\right)=\int_{X}DXP\left[X|I\right]\delta \left(X\left(t\right)-x\right).$$

By differentiating Equation (12) with respect to time, we obtain the continuity equation for the instantaneous probability density [12]:

(13) $$\begin{array}{cc} \frac{\partial \rho }{\partial t} & =\int_{X}DXP\left[X|I\right]\frac{\partial }{\partial t}\delta \left(X\left(t\right)-x\right)\\ & =\int_{X}DXP\left[X|I\right]\left(\nabla \delta (X(t)-x)\right)\cdot \dot{X}\left(t\right)\\ & =-\nabla_{x}\cdot \left(\int_{X}DXP\left[X|I\right]\delta \left(X\left(t\right)-x\right)\dot{X}\left(t\right)\right)\\ & =-\nabla_{x}\cdot \left(\rho \cdot v(x;t)\right), \end{array}$$

where v(x;t) is the velocity field that describes the flow of probability, given by

(14) $$v\left(x;t\right)={\langle \dot{X}\left(t\right)\rangle }_{X(t)=x,I}=\frac{1}{\rho (x;t)}{\langle \dot{X}\left(t\right)\delta \left(X\left(t\right)-x\right)\rangle }_{I}.$$

This equation describes the global and local conservation of the probability of finding the system in a given state x at a time *t*, and is guaranteed to hold for any system moving continuously in time through paths X(t)∈X. In the same way, it is possible to derive a dynamical equation for the velocity field v(x;t) itself, by differentiating it with respect to time. We have:

(15) $$\begin{array}{cc} \frac{\partial }{\partial t}\left(\rho \cdot v_{\mu }\right) & =\frac{\partial }{\partial t}\int_{X}DXP\left[X|I\right]\delta \left(X\left(t\right)-x\right)\dot{X_{\mu }}\left(t\right)\\ & =\int_{X}DXP\left[X|I\right]\frac{\partial }{\partial t}\delta \left(X\left(t\right)-x\right)\dot{X_{\mu }}\left(t\right)\\ & =\int_{X}DXP\left[X|I\right]\left(\delta \left(X\left(t\right)-x\right)\ddot{X_{\mu }}\left(t\right)+\partial_{\nu }\delta \left(X\left(t\right)-x\right)\dot{X_{\mu }}\left(t\right)\dot{X_{\nu }}\left(t\right)\right)\\ & ={\langle \delta \left(X\left(t\right)-x\right)\ddot{X_{\mu }}\left(t\right)\rangle }_{I}-\partial_{\nu }{\langle \delta \left(X\left(t\right)-x\right))\dot{X_{\mu }}\left(t\right)\dot{X_{\nu }}\left(t\right)\rangle }_{I}\\ & =\rho \;a_{\mu }\left(x;t\right)-\partial_{\nu }\left(\rho \;[C_{\mu \nu }+v_{\mu }v_{\nu }]\right), \end{array}$$

where in the last line we have defined the acceleration field as

(16) $$a\left(x;t\right):={\langle \ddot{X}\left(t\right)\rangle }_{X(t)=x,I}$$

and the velocity covariance matrix

(17) $$C_{\mu \nu }\left(x;t\right)={\langle {\dot{X}}_{\mu }\left(t\right){\dot{X}}_{\nu }\left(t\right)\rangle }_{X(t)=x,I}-v_{\mu }\left(x;t\right)v_{\nu }\left(x;t\right).$$

By using the continuity equation (Equation (13)) to rewrite the left-hand side as

(18) $$\begin{array}{cc} \frac{\partial }{\partial t}\left(\rho \cdot v_{\mu }\right) & =\frac{\partial \rho }{\partial t}v_{\mu }+\rho \frac{\partial v_{\mu }}{\partial t}\\ & =-\nabla_{x}\cdot \left(\rho \cdot v\right)v_{\mu }+\rho \frac{\partial v_{\mu }}{\partial t}\\ & =-\partial_{\nu }\left(\rho v_{\nu }\right)v_{\mu }+\rho \frac{\partial v_{\mu }}{\partial t}, \end{array}$$

we obtain, dividing both sides by ρ and using Equation (15), that

(19) $$\frac{\partial v_{\mu }}{\partial t}-\left(\partial_{\nu }v_{\nu }\right)v_{\mu }-v_{\nu }v_{\mu }\left(\partial_{\nu }\ln\rho \right)=a_{\mu }-\partial_{\nu }\left(C_{\mu \nu }+v_{\mu }v_{\nu }\right)-\left(C_{\mu \nu }-v_{\mu }v_{\nu }\right)\partial_{\nu }\ln\rho .$$

The term $v_{\nu }v_{\mu }\partial_{\nu }\ln\rho$ cancels, and we have:

(20) $$\frac{\partial v_{\mu }}{\partial t}-\left(\partial_{\nu }v_{\nu }\right)v_{\mu }=a_{\mu }-\partial_{\nu }C_{\mu \nu }-\partial_{\nu }\left(v_{\mu }v_{\nu }\right)-C_{\mu \nu }\partial_{\nu }\ln\rho ,$$

from which we now cancel the term $\left(\partial_{\nu }v_{\nu }\right)v_{\mu }$, arriving at

(21) $$\frac{\partial v_{\mu }}{\partial t}=a_{\mu }-\partial_{\nu }C_{\mu \nu }-v_{\nu }\partial_{\nu }v_{\mu }-C_{\mu \nu }\partial_{\nu }\ln\rho .$$

Rearranging the derivatives of $v_{\mu }$ in the left-hand side, we have

(22) $$\begin{array}{cc} \left(\frac{\partial }{\partial t}+v_{\nu }\partial_{\nu }\right)v_{\mu } & =a_{\mu }-\partial_{\nu }C_{\mu \nu }-C_{\mu \nu }\partial_{\nu }\ln\rho \\ & =a_{\mu }-\frac{1}{\rho }\partial_{\nu }\left(\rho \;C_{\mu \nu }\right)\\ & =a_{\mu }+\frac{1}{\rho }\partial_{\nu }\sigma_{\mu \nu }, \end{array}$$

which is the Cauchy momentum equation

(23) $$\left(\frac{\partial }{\partial t}+v\cdot \nabla \right)v=\frac{Dv}{Dt}=a\left(x;t\right)+\frac{1}{\rho }\nabla \cdot \overset{↔}{\sigma },$$

with D/Dt the *convective derivative* and $\overset{↔}{\sigma }=-\rho \cdot \overset{↔}{C}$ the stress tensor.

Equations (13) and (23) form a closed coupled system of equations for ρ(x;t) and v(x;t), needing as their only external input the velocity covariance matrix $C_{\mu \nu }$. These equations are then *built-in features* of inference over paths. In a Bayesian approach, they are valid for any system that moves continuously in time. The Cauchy momentum equation includes most notably the Navier–Stokes equation as a particular case [13].

## 5. Including Particular Knowledge into Our Models

At this point, we have developed a generic framework where no particular details about a system have been included. Clearly all those details have to be contained in P[X|I], or rather, in the covariance matrix $C_{\mu \nu }\left(x;t\right)$ which can be derived from it. The question remains about how to incorporate these details in the most unbiased manner. In principle, we could start from the *null* assumption of equiprobable paths,

$$P[X|I_{0}]=\mathrm{constant},$$

and add new information R later on, by updating our probability functional $P[X|I_{0}]$ to a new P[X|I], where $I=(I_{0},R)$. There are essentially two equivalent methods to achieve this, and depending on the actual form of R, one of them may be more directly applicable than the other.

(1) **Bayes’ theorem**: the posterior distribution $P(u|I_{0},R)$ is given in terms of the prior $P(u|I_{0})$ by
  $$P\left(u|I_{0},R\right)=\frac{P\left(u|I_{0}\right)\cdot P\left(R|u,I_{0}\right)}{P(R|I_{0})}.$$
  This method is most useful when R is comprised of statements about the states u (e.g., boundary conditions).
(2) **Principle of maximum entropy**: the posterior distribution p(u) is the one that maximizes
  $$S\left[p_{0}\to p\right]=-\int_{V}du\;p\left(u\right)\ln\frac{p(u)}{p_{0}\left(u\right)},$$
  where $p_{0}\left(u\right)$ is the prior distribution. This method is most useful when R consists of constraints on the final model p(u), usually expressed as fixed expected values.

In Reference [2], Jaynes assumes the continuity equation from the start and derives the flux J(x;t)=ρ·v(x;t) from symmetry considerations, the central limit theorem, and Bayes theorem. In our classification, this corresponds to method (1). In Reference [5], Gull recovers Brownian motion by essentially performing discrete-time maximum caliber inference under constraints over location and particle speed, hence corresponding to an application of method (2).

## 6. The Maximum Caliber Principle

The function p(u) that is closest to our prior probability $p_{0}\left(u\right)$ and is consistent with the constraints R is the one that maximizes the relative entropy [14,15]

$$S\left[p_{0}\to p\right]=-\int_{V}dup\left(u\right)\ln\frac{p(u)}{p_{0}\left(u\right)}$$

among the set of functions *p* that are compatible with R. The negative of this relative entropy, known as the Kullback–Leibler divergence, is commonly used to measure the “informational distance” from $p_{0}$ to *p*. It is important to note that this is a rule of inference and not a physical principle, and therefore it is not bounded by the meaning assigned to the states x, as long as we can write (Bayesian) probabilities over them.

For the general case of *m* constraints of the form

$$\langle f_{j}\left(u\right)\rangle =F_{j},\;\;\;\;\;\;\;\;\left(\;j=1,\ldots ,m\right),$$

the maximum entropy solution starting from $P(u|I_{0})$ is obtained through the use of *m* Lagrange multipliers (one for each constraint),

(24) $$P\left(u|I_{0},R\right)=P\left(u|I_{0}\right)\left[\frac{\exp\left(-\sum_{j=1}^{m}\lambda_{j}f_{j}\left(x\right)\right)}{Z(\lambda )}\right],$$

where Z(λ) is the partition function. This is compatible with Bayesian updating, as this posterior distribution is proportional to the prior. The Lagrange multipliers are solutions of the constraint equations in terms of *Z*:

(25) $$F_{j}=\frac{\partial }{\partial \lambda_{j}}\ln Z\left(\lambda \right)\;\;\;\;\;\;\;\;\left(\;j=1,\ldots ,m\right).$$

In exactly the same way, the path (relative) entropy (sometimes known as the *caliber*) is defined as the path integral [1,16,17,18,19,20,21,22]:

$$S\left[p_{0}\to p\right]=-\int_{X}DX\;p\left[X\right]\ln\frac{p[X]}{p_{0}\left[X\right]},$$

where $p_{0}\left[X\right]:=P\left[X|I_{0}\right]$ is the prior path probability. The use of this generalization is justified based on the fact that we can write any path X(t) in terms of a complete orthonormal basis $\left\{B_{i}\right\}$,

(26) $$X\left(t\right)=\sum_{n=0}^{N-1}\gamma_{n}B_{n}\left(t\right),\;\;\;\;\;\;\;\;N\to \infty ,$$

and then there is a one-to-one correspondence between every path X(t) and its coordinates $\left(\gamma_{0},\gamma_{1},\ldots ,\gamma_{N-1}\right)$. Inference over paths X then becomes completely equivalent to inference over the coefficients γ, which form a system with *N* degrees of freedom.

In summary, for the general maximum caliber inference problem we have *m* constraints, written as

(27) $$\langle F_{j}\left[X\right]\rangle =F_{j}\;\;\;\;\;\;\;\;\left(\;j=1,\ldots ,m\right),$$

from which the probability functional obtained is

(28) $$P\left[X|I_{0},R\right]=\frac{1}{Z(\lambda )}P\left[X|I_{0}\right]\exp\left(-\sum_{j=1}^{m}\lambda_{j}F_{j}\left[X\right]\right).$$

Any such maximum caliber solution can be cast in the “canonical” form, as

(29) $$P\left[X|I\right]=\frac{1}{Z(\alpha )}\exp\left(-\frac{1}{\alpha }A\left[X\right]\right),$$

where A is a functional, analogous to the Hamilton action of a classical system, and α>0 is a constant with the same physical units as A. By simple inspection of this canonical form, it is straightforward to see that the most probable path is the one with minimum action:

(30) $$\frac{\delta A[X]}{\delta X(t)}=0,\;\;\;\;\forall t.$$

## 7. An Illustration: Newtonian Mechanics of Charged Particles

As an example of the application of this formalism, consider a “particle” with known square speed $\nu^{2}$, known instantaneous probability density ρ, and known velocity field v(x;t) for all times t∈[0,τ]. The corresponding constraints are then

(31) $$\begin{array}{c} \langle |\dot{X}\left(t\right){|}^{2}\rangle =\nu^{2}\left(t\right)\;\;\;\;\;\;\;\forall t, \end{array}$$

(32) $$\begin{array}{c} \langle \delta (X(t)-x)\rangle =\rho \left(x;t\right)\;\;\;\;\;\;\;\forall x,t, \end{array}$$

(33) $$\begin{array}{c} \langle \dot{X}\left(t\right)\delta \left(X\left(t\right)-x\right)\rangle =\rho \left(x;t\right)v\left(x;t\right)\;\;\;\;\;\;\;\forall x,t. \end{array}$$

The resulting maximum caliber solution is of the form

(34) $$P\left[X|I\right]=\frac{1}{Z(\alpha )}\exp\left(-\frac{1}{\alpha }\int_{0}^{\tau }dt\;L\left(X\left(t\right),\dot{X}\left(t\right);t\right)\right),$$

with the Hamilton action

(35) $$A\left[X\right]=\int_{0}^{\tau }dtL\left(X\left(t\right),\dot{X}\left(t\right);t\right),$$

and a Lagrangian defined as

(36) $$L\left(X\left(t\right),\dot{X}\left(t\right);t\right)=\lambda_{1}\left(t\right){\left|\dot{X}\left(t\right)\right|}^{2}+\int_{V}dx\left(\lambda_{2}\left(x;t\right)+\lambda_{3}\left(x;t\right)\cdot \dot{X}\left(t\right)\right)\delta \left(X\left(t\right)-x\right),$$

where $\lambda_{1}$, $\lambda_{2}$, and $\lambda_{3}$ are Lagrange multipliers. This Lagrangian can be cast into a more familiar form,

(37) $$L\left(X\left(t\right),\dot{X}\left(t\right);t\right)=\frac{1}{2}m\left(t\right)\dot{X}{\left(t\right)}^{2}-\Phi \left(X\left(t\right);t\right)+\dot{X}\left(t\right)\cdot A\left(X\left(t\right);t\right),$$

by simply renaming the Lagrange multipliers and integrating the delta function [18]. Interestingly, this is none other than the Lagrangian for a particle with time-dependent “mass” m(t) in an external “electromagnetic potential” (Φ,A). The most probable path under these constraints is determined by the solution of the Euler–Lagrange equation, which reduces to Newton’s second law under a “Lorenz force”,

(38) $$\frac{d}{dt}\left(mv\right)=-\nabla \Phi \left(x;t\right)-\frac{\partial A(x;t)}{\partial t}+v\times \left(\nabla \times A\left(x;t\right)\right),$$

as shown in the Appendix. In particular, it is important to note that it is the constraint on the squared speed $\nu^{2}\left(t\right)$ that adds the mass m(t) to the model, as $m=2\lambda_{1}$, the constraint on the probability density ρ(x;t) adds the scalar potential Φ(x;t) to the model, as $\Phi =-\lambda_{2}$, and finally the constraint on the local velocity field v(x;t) adds the vector potential A(x;t) to the model, because $A=\lambda_{3}$. Nowhere in the derivation of this Lagrangian have we assumed the existence of charges, electromagnetic fields, or the Lorenz force. The structure that is revealed is the most unbiased under the constraints given in Equations (31) to (33), that is, with approximate knowledge of its location (given by ρ) and velocity “field lines” (given by v). This model could be used for people in a busy street crossing, or vehicles in a city.

## 8. Concluding Remarks

We have shown that it is possible to construct a fluid theory from Bayesian inference of an abstract system with *N* degrees of freedom moving along paths X(t), and that this theory automatically includes the continuity equation and the Cauchy momentum equation as built-in features. Moreover, through the use of the Maximum Caliber principle, it is possible to formulate the dynamics of such an abstract system in terms of an action that is minimal for the most probable path, resembling the well-known structures of Lagrangian and Hamiltonian mechanics.

By entering the square speed, instantaneous probability density, and velocity field into our model, a Lagrangian of a “particle” under external fields emerges naturally. This “particle” moves on average according to Newton’s law of motion F=m·a under the Lorenz force, with scalar and vector potentials determined by the known information about the location and velocity lines. In this formulation, the only ingredients that we could call *physical* were the existence of an *N*-dimensional “particle” moving continuously along (unknown) paths. In this application, we see that position and velocity are the only intrinsic (*real* or *ontological*) properties of the particle at a given time *t*. On the other hand, the time-dependent mass m(t) and the fields Φ(x;t) and A(x;t) are emergent parameters (in fact, Lagrange multipliers) needed to impose the constraints on the known information used to construct the model.

## Appendix A. Derivation of the Lorenz Force from the Lagrangian of a Particle in an Electromagnetic Field

We start from the Euler–Lagrange equation

(A1) $$\frac{\partial L}{\partial x_{\mu }}-\frac{d}{dt}\left(\frac{\partial L}{\partial v_{\mu }}\right)=0$$

for the Lagrangian in Equation (37),

$$L\left(x,v;t\right)=\frac{1}{2}m\left(t\right)v^{2}-\Phi \left(x;t\right)+v_{\nu }A_{\nu }\left(x;t\right),$$

which gives

(A2) $$-\partial_{\mu }\Phi +v_{\nu }\partial_{\mu }A_{\nu }-\frac{d}{dt}\left(mv_{\mu }+A_{\mu }\right)=0.$$

Replacing the total time derivative of the vector field A(x;t),

$$\frac{d}{dt}A_{\mu }\left(x;t\right)=\partial_{t}A_{\mu }+v_{\nu }\partial_{\nu }A_{\mu },$$

we have

(A3) $$\frac{d}{dt}\left(mv_{\mu }\right)+\partial_{t}A_{\mu }+v_{\nu }\partial_{\nu }A_{\mu }=-\partial_{\mu }\Phi +v_{\nu }\partial_{\mu }A_{\nu },$$

which can be written as

(A4) $$\frac{d}{dt}\left(mv_{\mu }\right)=\left(-\partial_{\mu }\Phi -\partial_{t}A_{\mu }\right)+v_{\nu }\left(\partial_{\mu }A_{\nu }-\partial_{\nu }A_{\mu }\right).$$

Using the fact that the *i*-th component of v×(∇×A) is given by

(A5) $$\begin{array}{cc} {\left[v\times (\nabla \times A)\right]}_{i} & =\epsilon_{ijk}v_{j}\left(\epsilon_{klm}\partial_{l}A_{m}\right)\\ & =\epsilon_{ijk}\epsilon_{klm}\left(v_{j}\partial_{l}A_{m}\right)\\ & =\left(\delta_{il}\delta_{jm}-\delta_{im}\delta_{jl}\right)v_{j}\partial_{l}A_{m}\\ & =v_{j}\left(\partial_{i}A_{j}-\partial_{j}A_{i}\right), \end{array}$$

we finally obtain

(A6) $$\frac{d}{dt}\left(mv_{\mu }\right)=\left(-\partial_{\mu }\Phi -\partial_{t}A_{\mu }\right)+{\left[v\times (\nabla \times A)\right]}_{\mu },$$

which is Equation (38).
